# Autopsy of Alexander III, Emperor of Russia

**Published:** 1895-02

**Authors:** 


### AUTOPSY OF ALEXANDER III, EMPEROR OF RUSSIA.

TRANSLATED BY FREDERIC PRESTON, M. D., CHESTER, PA.

We give below the full text of the official diagnosis and autopsy of Alexander III.

Diagnosis :—Chronic interstitial nephritis followed by lesions of the heart and blood-vessels. Hemorrhagic infarctus of the left lung, followed by pneumonia.

LIVADIA, 21 Oct. (2 November), 1894.

Professors Leyden, Zakharine, and Popow, and Drs. Hirsch and Veliaminow, honorary surgeons of Court.

Autopsy :—In the year 1894 on the day of 22d October (3 Nov.) at 7.30 P. M., we the undersigned proceeding to the embalming of the body of the ex-emperor have noted the following pathologic changes :

Œdema considerable of the subcutaneous tissue of the lower extremities and reddish spots on left knee. The cavity of the left pleura contained 280 CC of serous reddish liquid. The cavity of right pleura contained 50 CC of same liquid.

The summit of right lung bore a fibrous cicatrix of ancient date ; the right lung being œdematous. The superior lobe of left lung was slightly œdematous and there was an hemorrhagic infarctus in the inferior lobe, which was much congested with blood and contained little air. This infarctus was found on the superior border of the inferior lobe of left lung, and presented on section, the form of a triangle, one and one-half centimeters high, and one centimeter broad. The cavity of the pericardium contained 30 CC of serous red-tinted liquid.

The size of the heart was considerably increased ; the vertical diameter being 17 centimeters, and the horizontal diameter being 18 centimeters. The sub-serous tissue contained a great quantity of fatty matter.

The heart was found in a condition of slight diastole, the left cavity being enlarged and the wall of left ventricle thickened (2 centimeters). The muscular tissue of left ventricle was pale, flabby, and of a yellowish tint. In the right ventricle the muscular wall was thinned (6 millimeters), and of the same yellow tint. The valvular apparatus was in a completely normal condition.

The abdominal cavity contained 200 CC of serous liquid. The stomach and intestines contained a great quantity of gas. The volume of the liver was slightly increased, and the organ very plethoric (congested ?). The kidneys had the following dimensions :

Left, 16 centimeters long ; 7 centimeters broad, and 4 centimeters thick.

Right, 15 centimeters long ; 6½ centimeters broad, and 4 centimeters thick.

The capsules of the kidneys were of normal thickness, and easily detachable. The surface of the kidneys was slightly granulated, and of a dark red color, the hardening of the kidneys was insignificant.

The cortical substance of the kidneys was lessened (from 6 to 7 millimeters), and of a yellowish tint ; the medullary substance, of dark red color. There was also found in the left kidney a serous cyst 3 millimeters in diameter.

In virtue of the preceding facts we formulate as our opinion, that his Majesty the Emperor Alexander Alexandrovitch succumbed to a cardiac paralysis, preceded by a muscular degeneration of a hypertrophied heart, and of an interstitial nephritis (granular atrophy of kidneys).

LIVADIA, 22 October (3 November), 1894.

Signed. Professors F. J. Klein, D. F. Zernow, M. A. Popow, and Drs. A. V. Altonkhow and A. C. Beloussow.—*Semaine Medicale*, November, 1894.

